# Structural, microstructural and magnetic evolution in cryo milled carbon doped MnAl

**DOI:** 10.1038/s41598-018-20606-8

**Published:** 2018-02-06

**Authors:** Hailiang Fang, Johan Cedervall, Daniel Hedlund, Samrand Shafeie, Stefano Deledda, Fredrik Olsson, Linus von Fieandt, Jozef Bednarcik, Peter Svedlindh, Klas Gunnarsson, Martin Sahlberg

**Affiliations:** 1 Department of Chemistry – Ångström Laboratory, Uppsala University, Box 538, 75121 Uppsala, Sweden; 2 Department of Engineering Sciences, Uppsala University, Box 534, 751 21 Uppsala, Sweden; 3 Deutsches Elektronen Synchrotron DESY, Notkestrasse 85, D-22603 Hamburg, Germany; 4 Institute for Energy Technology Instituttveien, 18NO-2007 Kjeller, Norway; 5 Höganäs AB, Bruksgatan 35, 263 33 Höganäs, Sweden

## Abstract

The low cost, rare earth free τ-phase of MnAl has high potential to partially replace bonded Nd_2_Fe_14_B rare earth permanent magnets. However, the τ-phase is metastable and it is experimentally difficult to obtain powders suitable for the permanent magnet alignment process, which requires the fine powders to have an appropriate microstructure and high τ-phase purity. In this work, a new method to make high purity τ-phase fine powders is presented. A high purity τ-phase Mn_0.55_Al_0.45_C_0.02_ alloy was synthesized by the drop synthesis method. The drop synthesized material was subjected to cryo milling and  followed by a flash heating process. The crystal structure and microstructure of the drop synthesized, cryo milled and flash heated samples were studied by X-ray *in situ* powder diffraction, scanning electron microscopy, X-ray energy dispersive spectroscopy and electron backscatter diffraction. Magnetic properties and magnetic structure of the drop synthesized, cryo milled, flash heated  samples were characterized by magnetometry and neutron powder diffraction, respectively. The results reveal that the 2 and 4 hours cryo milled and flash heated samples both exhibit high τ-phase purity and micron-sized round particle shapes. Moreover, the flash heated samples display high saturation magnetization as well as increased coercivity.

## Introduction

Permanent magnets play a crucial role in advanced green energy technologies like wind turbines, electric and hybrid cars^1^. However, the market for permanent magnets mainly consists of Nd_2_Fe_14_B and ferrites, where the former not only contain Nd but also Dy and other rare earth elements as additives^2^. The supply of some heavy rare earth elements like Dy, Tb and Sm for high temperature application permanent magnets is quite limited and large fluctuations in rare earth price has occurred. Thus, it is predicted that these elements will have supply shortage according to current consumption rate in the coming decades if no alternative materials made from more abundant elements are found^3^.

Mn-based magnetic materials like MnAl, MnGa and MnBi, on the other hand, provide a combination of large magnetocrystalline anisotropy, high Curie temperature and a maximum energy product (*BH*)_*max*_ between ferrite and rare earth based magnets and have therefore received more attention lately^4–6^. In particular, MnAl based magnetic materials (~50–60 at.% Mn) with the *L1*_*0*_-type structure (τ-phase) have great potential to become a high performance permanent magnet materials at low cost (cost of raw materials ≈2 $/kg^7^), if appropriate processing route could be developed.

Off-stoichiometric Mn-rich compositions are needed to obtain ferromagnetic properties in MnAl magnetic materials. Theoretical results for the composition Mn_1.14_Al_0.86_ give a total magnetic moment of 1.98 μ_B_/f.u. (*M*_*s*_ = 0.69 MA/m), a large Curie temperature (670 K) and magnetic anisotropy energy as large as 2.18 MJ/m^3^ ^8^. Experimental results extrapolated to 0 K for Mn_0.54_Al_0.44_C_0.02_ show values of the saturation magnetization *M*_*s*_ = 0.68 MA/m and the magnetic anisotropy energy *K*_1_ = 1.7 MJ/m^3^ ^9^, in good agreement with theory^8^.

However, the τ-phase MnAl is metastable and is easily decomposed into thermodynamically more stable β-Mn and γ_2_-phases (Al_8_Mn_5_)^10,11^. Addition of carbon at the octahedral interstitial sites (½, ½, 0) have proven to be an effective way of stabilizing the tetragonal structure with an elongation along the *c* *axis*^11,12^. Previous studies by us have also shown the importance of carbon doping on the stability of the τ-phase^13,14^ and in the present study only carbon doped samples were used. Carbon furthermore has an effect of increasing the saturation magnetization but reduces the Curie temperature and the anisotropy^12,15^.

Previous research show that the τ-phase is formed through a two-step process, originating from the parent hexagonal ɛ-phase that transforms into the intermediate B19-structure ɛ’-phase which in turn transforms into the τ-phase if sufficient undercooling is achieved at 723 K < T < 823 K^16,17^. These transformations are believed to occur independently of the composition and are controlled by nucleation and growth processes^17^. It has been shown that the transformation is highly dependent on the nucleation of the τ-phase at the interphase with the ɛ-phase^16^.

Due to the importance of the microstructure on the magnetic properties several processes have been reported for the fabrication of the τ-phase, including nanocrystalline powder from mechanical milling^12,18^, melt spinning followed by cryo milling^19^; however, most of the reported processing methods are either complex or the final properties are not sufficiently good (*e*.*g*., the (*BH*)_*max*_ is too low) for implementation in industrial permanent magnet applications.

The (*BH*)_*max*_ performance of a permanent magnet not only depends upon the intrinsic properties like (*M*_*s*_, *K*_1_ and *T*_*c*_) of the compounds, but is also tightly related to external factors like microstructure of the magnet, grain size and texture orientation^1^. This means that the consisting grains of the magnets need to align along a specific direction to have a maximum (*BH*)_*max*_ value^20^.

There are mainly two approaches to prepare the textured MnAl magnets. The first approach is through high temperature extrusion like hot deformation or compaction, various work through this method have been reported previously^21,22^. The second approach is through ball/surfactant milling of the gas atomized or induction/arc melted sample followed by magnetic field compaction^23^. The former method is too expensive and the latter one produces flakes with random crystallographic orientation due to the large ductility of MnAl, thus impeding proper orientation of the grains in a magnetic field^13,23–25^. Consequently, the preparation of powders consisting of regularly shaped single grain particles would be critically important for the success of a high performance anisotropic permanent magnet material^26^.

In this paper it is reported: *i*) the use of cryo milling of drop synthesized high purity Mn_0.55_Al_0.45_C_0.02_ (τ -phase) to prepare powder with small particle sizes ~20 µm and rounded shapes; *ii*) the evolution of the τ → ɛ → τ as a function of the heating/cooling rate and annealing temperature using *in situ* synchrotron radiation on high purity cryo milled τ-phase powder; *iii*) the correlation between cryo milling time and the evolution of the microstructure, crystal structure and Mn/Al ordering with the magnetic properties using light optical microscopy (LOM), scanning electron microscopy (SEM) with X-ray energy dispersive spectroscopy (EDS), electron backscatter diffraction (EBSD), and magnetometry (M); and *iv*) the effect of short annealing times at 900 °C with fast cooling (flash heating procedure) to regain the highly crystalline τ-phase within the individual grains, to maximize *M*_*s*_ and *H*_*c*_, and to facilitate the ideal condition of one grain per one powder particle for making a high performance MnAl permanent magnet. The results provide new insight into the effect of the milling process, and how the negative effects from milling (*e*.*g*., site intermixing of Mn and Al) on the magnetic properties can be largely reversed using our proposed flash heating method.

## Results

### X-ray and neutron powder diffraction

**Figure 1:**
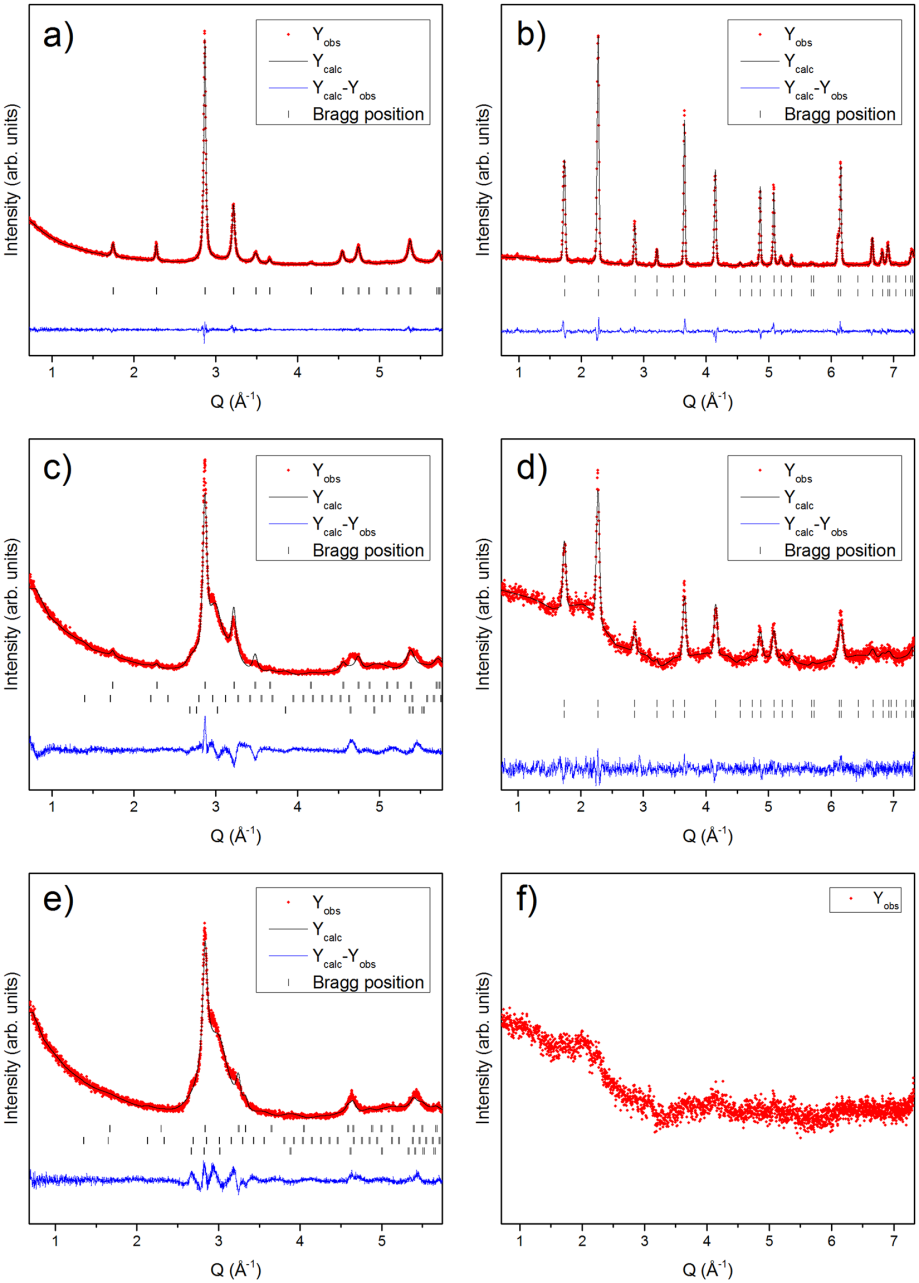
Refined powder diffraction data of (**a**,**b**) DS, (**c**,**d**) 2CM) and (**e**,**f**) 4CM. The XRPD and the NPD data are presented on the left and right side, respectively. For the X-ray diffraction figure in (**a**), the peak positions of the τ-phase; (**c**); (**e**), the peak positions of the τ-phase (upper), β-phase (middle) and ɛ-phase (lower) are shown below the patterns as vertical bars. For the neutron diffraction figure in (**b**); (**d**), the peak positions contributed from the atomic structure (upper), and magnetic structure (lower) are shown below the patterns as vertical bars.

The refined powder diffraction data of drop synthesized (DS), 2 h cryo milled (2CM) and 4 h cryo milled (4CM) samples are shown in Fig. 1. From the XRPD data (Fig. 1a,c,e) a clear decrease in the peak intensities combined with a pronounced peak width broadening is observed with longer milling time. In addition, several of the weaker peaks (i.*e*., 1.73, 2.27 and 3.47 Å^−1^) related to the τ-phase (*i*.*e*., the (001) and the (100) and the (002) planes), gradually disappear with longer milling time. However, the remaining strong reflections (*i*.*e*., between 2.7 Å^−1^ ≤ Q ≤ 3.5 Å^−1^) from XRPD indicate that a crystalline phase is still preserved. On the contrary, the NPD data (Fig. 1b,d,f) show a strong decrease of the reflection intensities for the 2CM sample, while no reflections are observed for the 4CM sample (Fig. 1f), reminiscence of an amorphous phase.

The combined XRPD and NPD data (Fig. 1) of the DS, 2CM and 4CM samples were used to refine the lattice parameters in the space group *P4*/*mmm*. The joint refinement using both X-ray and neutron diffraction intensities was necessary to be able to refine the occupancy of the Mn and Al-sites. Reliability factors (R-values and χ^2^) for all refinements are listed in the supplementary information Tables SI1 and SI2. The NPD data was, however, not refined for the 4CM due to the lack of peaks. From Table 1 with data obtained from the Rietveld refinement of the combined XRPD and NPD data, the occupancy of the Mn and Al-sites are found to vary with increased milling time. It is found that the Mn content at the Mn 1a (0, 0, 0) site decreases from 94% to 75%, while the Al content increases from 6% to 25% after 2 hours of cryo milling. The opposite is observed at the Al 1d (½, ½, ½) site, where the Al content decreases from 85% to 66% after 2 hours of cryo milling (Table 1).

**Table 1:** Refined atomic occupancies for the DS, 2CM, 4CM and the flash heated samples.

samples	Atom	Site occupancy (%)	
		1a(0, 0, 0)	1d(1/2, 1/2, 1/2)
Drop sythesized	Mn	94.8(8)	14.5(8)
	Al	5.2(2)	85.4(2)
2 hours CM	Mn	75.4(6)	33.5(7)
	Al	24.6(4)	66.4(3)
4 hours CM	Mn	N/A	N/A
	Al	N/A	N/A
Flash heated (all)	Mn	95	15
	Al	5	85

**Figure 2:**
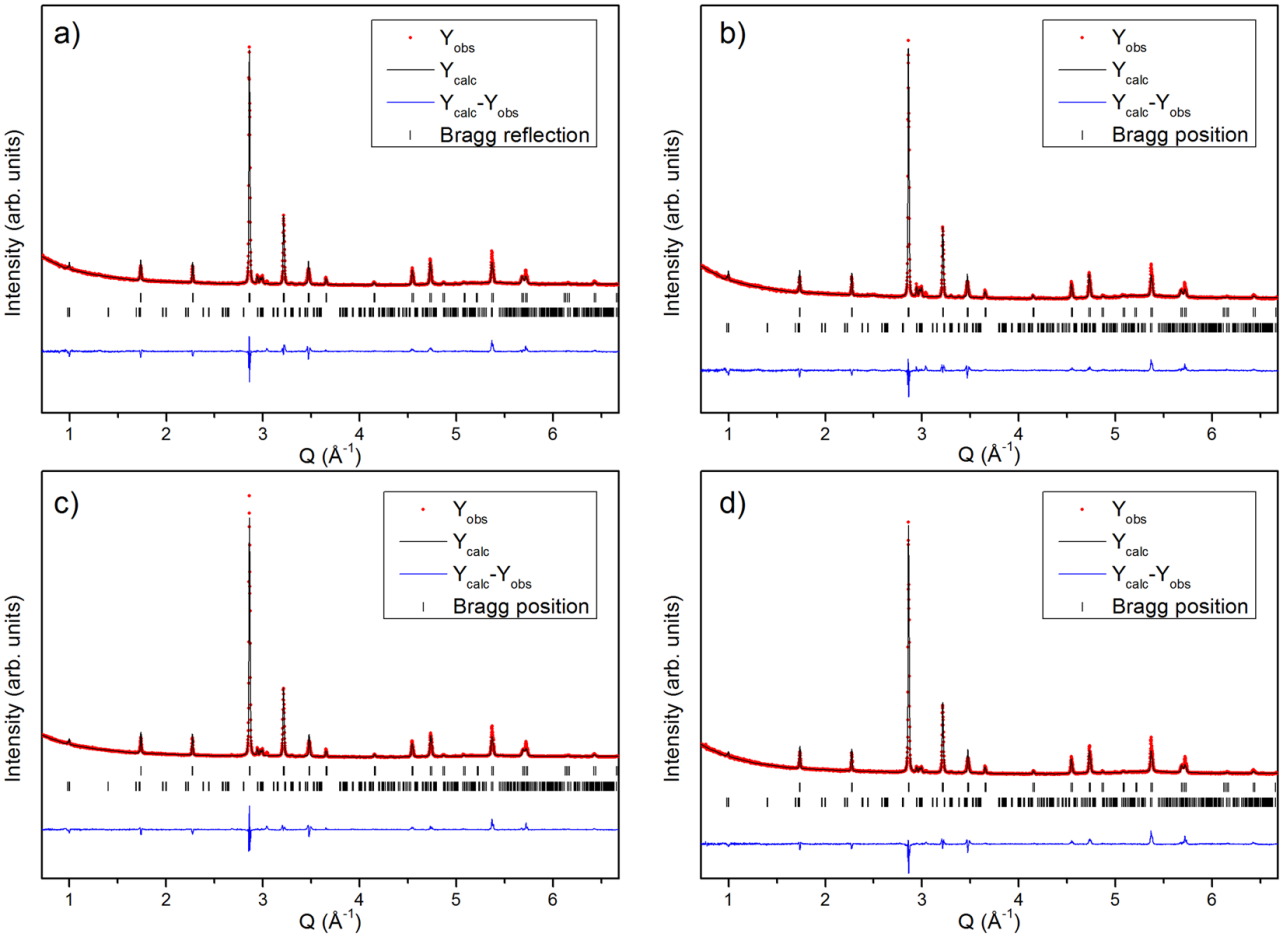
Refined XRPD of cryo milled and flash heated Mn_0.55_Al_0.45_C_0.02_ samples. (**a**) 2 hours cryo milled and 900 °C + 5 minutes flash heated; (**b**) 2 hours cryo milled and 900 °C + 15 minutes flash heated; (**c**) 4 hours cryo milled and 900 °C + 5 minutes flash heated; (**d**) 4 hours cryo milled and 900 °C + 15 minutes flash heated. The peak positions of the τ-phase (upper) and γ_2_-phase (lower) are shown below the patterns as vertical bars (λ_1_ = 1.540600 Å, λ_2_ = 1.544390 Å).

Figure 2 shows refined XRPD patterns of the flash heated 2CM and 4CM powders. It is clearly observed that the flash heating process recrystallizes the powder significantly after only 5 min (Fig. 2a,c) to produce peaks comparable to the original DS sample (Fig. 1a). No difference is observed between the flash heated 2CM and 4CM XRPD patterns. All the XRPD patterns for the flash heated samples contain detectable amounts of the γ_2_-phase (Fig. 2) that are mainly observed ~3 Å^−1^, but the amount of these phases is quite low (<10%). The structural model used (with the same Mn and Al occupancies as in the DS sample) indicates that a reordering of the Mn and the Al on the two crystallographic sites takes place upon the heat treatment (Table 1).

**Figure 3:**
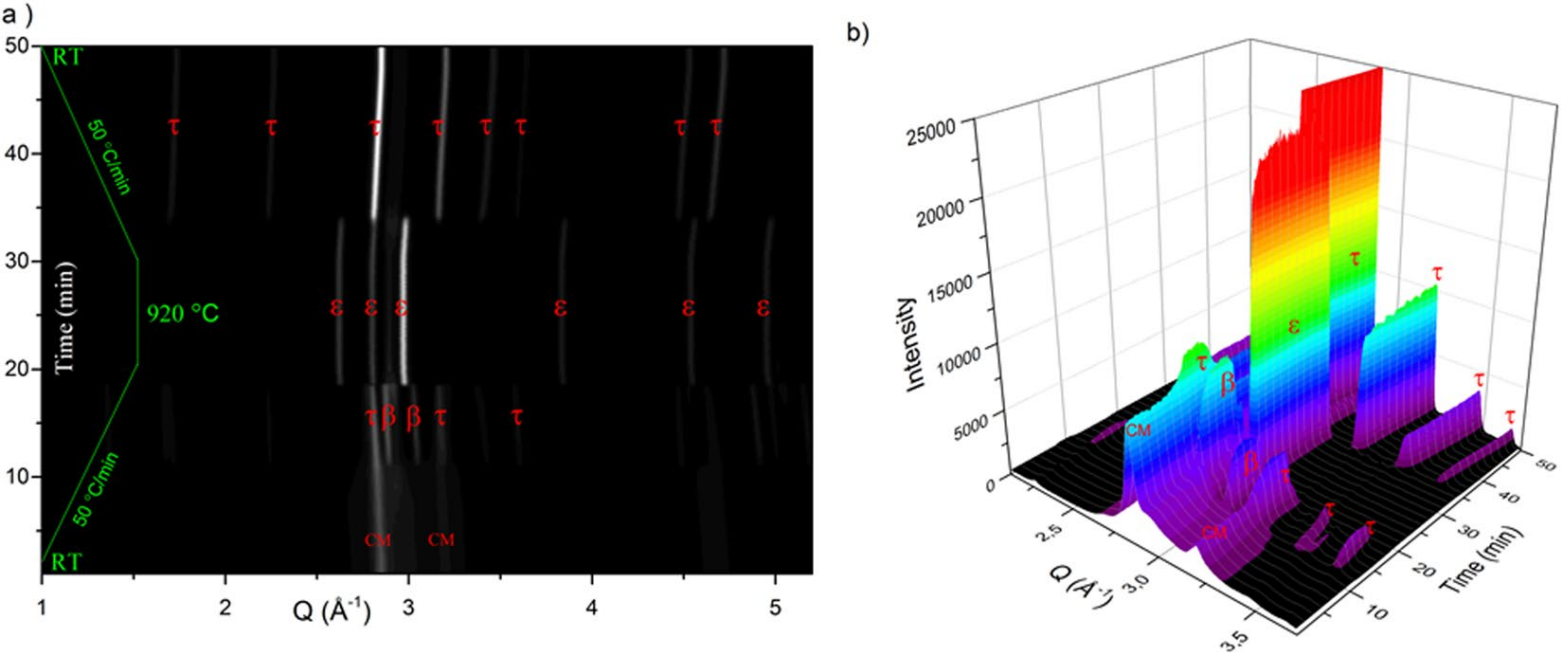
Densitometric view of the 2CM powder X-ray diffractograms (λ = 0.207 Å) recorded *in-situ* (where intensity is shown as a greyscale). (**a**) 1-D image (**b**) surface image.

To further investigate the stability range of the τ-phase as a function of heating rate and temperature, the 2CM sample was analyzed *in situ* by synchrotron radiation (λ = 0.207 Å). During the measurement, the powder was subjected to a heating rate of 50 °C/min from room temperature up to 920 °C and was kept for 5 minutes before being cooled down to room temperature again, with a rate of 50 °C/min (Fig. 3a,b). From Fig. 3a, it is observed that the 2CM powder decomposes into a mixture of β-Mn and τ-phase at ~500 °C. At *T* > 900 °C the powder transforms fully into pure ɛ-phase (Fig. 3a). However, during the cooling process at a cooling rate of 50 °C/min, pure τ-phase is reformed again at T < 830 °C.

Figure 3b shows the continuous change in the XRPD pattern during the *in situ* process. It is clear that the intensity of the τ-phase related peaks increase during the cooling process, and that the ɛ-phase fully transforms back to the τ-phase. The β-Mn phase that is formed at ~550–600 °C during heating process disappears at 900 °C when the τ → ɛ-phase transformation takes place. The β-Mn is, as expected, not observed to form during the cooling process (see Fig. 3a).

### Microstructural analysis

Local microstructure and composition has large implications on the final magnetic properties^27^. To further understand the variation of the local microstructure and composition in the DS sample, LOM, EBSD, SEM and EDS mapping was used.

**Figure 4:**
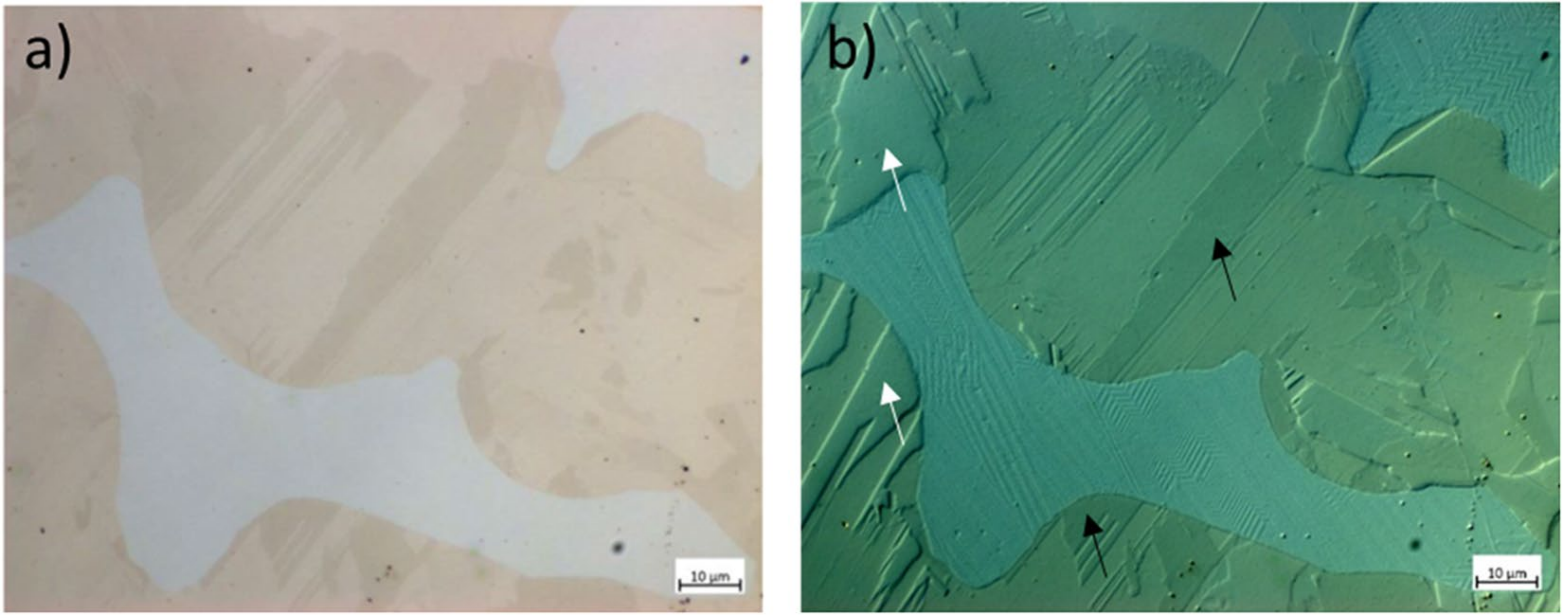
LOM Images of the DS sample using (**a**) bright-field and (**b**) C-DIC imaging. The black arrows indicate regions with similar crystallographic orientation, while the white arrows indicate regions of slightly different heights compared to the rest of the observed regions in the image.

From Fig. 4a, the bright-field image of the DS sample shows darker (beige) and lighter (white) regions. The corresponding Circular Differential Interference Contrast (C-DIC) image of the same region is presented in Fig. 4b where the orientations of the grains are more clearly observed. The black arrows in Fig. 4b indicate regions with similar crystallographic orientation, while the white arrows indicate regions of slightly different heights compared to the rest of the observed regions in the image. From Fig. 4 it can be concluded that the two regions observed within the beige (green) regions, consists of smaller grains aligned along different crystallographic orientations.

**Figure 5:**
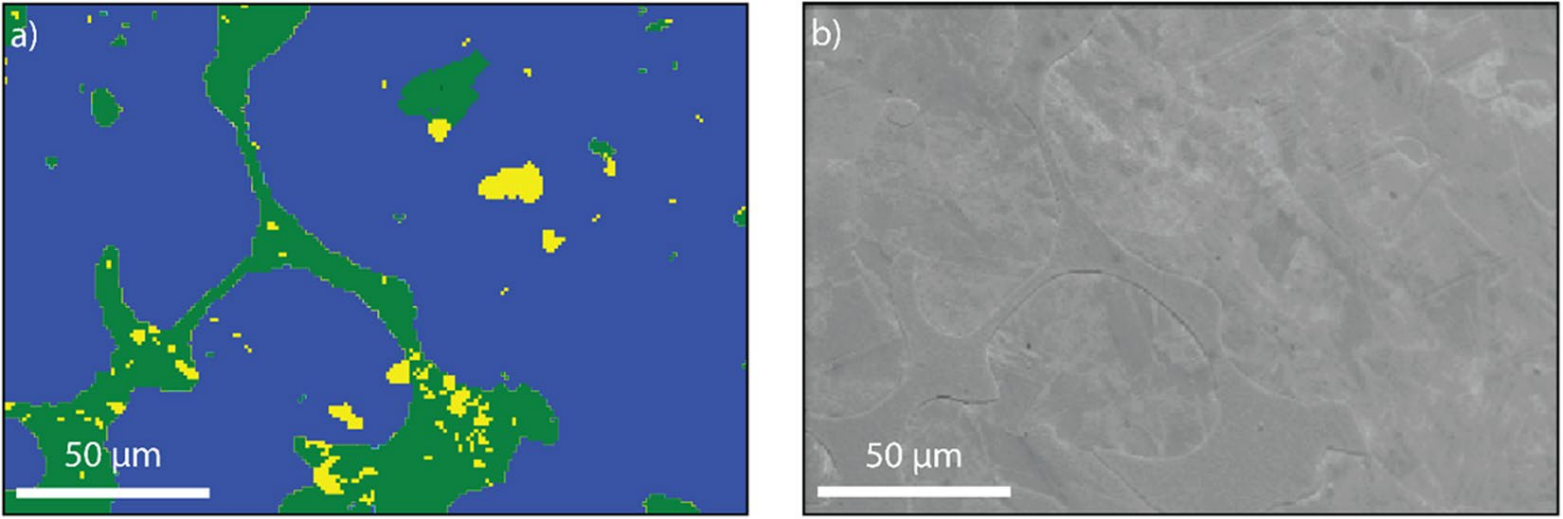
The DS sample as observed from (**a**) EBSD with the corresponding (**b**) SEM image from the same region.

From the EBSD analysis of the DS sample (Fig. 5), it is observed that the sample consists of one main phase (blue in Fig. 5a) and a secondary region (green in Fig. 5a) that resides at the boundaries of the blue phase. In Fig. 5a, the blue and green phases has been attributed to the τ-, and the ɛ-phase, respectively (see Figure SI1 for examples of diffraction patterns). However, the green region has been averaged (smoothed) in order to improve the visibility of the majority ɛ-phase, but locally consists of additional unidentified phases (unindexed patterns) in addition to the ɛ-phase (called “matrix region”). The different regions are also clearly distinguished in the SEM image in Fig. 5b, with the τ-phase showing a more complex topography, while the matrix region is apparently smoother. In addition, the DS sample also contains minute amounts of β-Mn and γ_2_-phases, observed as yellow inclusions in Fig. 5a.

**Figure 6:**
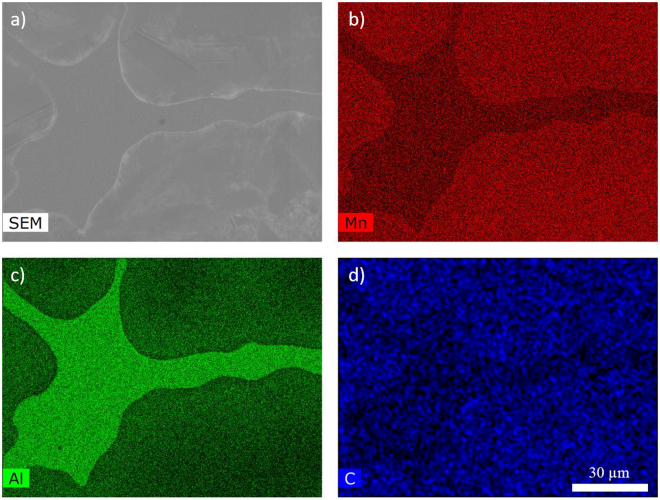
Analysis of the DS sample using (**a**) SEM and EDS mapping to show the (**b**) Mn-, (**c**) Al-, and (**d**) C-content in different regions.

To further understand the differences between the blue and the green regions (see Fig. 5a), EDS mapping was made on a polished DS sample. From Fig. 6 it is clear that the DS sample contains two distinct regions: 1) Mn-rich; and 2) Al-rich. It is clearly observed that the Al-rich phase preferentially enters or forms in the grain boundary regions (matrix region) in the DS sample. In addition, it is observed that the C-content is significantly lower in the Al-rich regions (see Fig. 6d), thus indicating that the Al-rich region is low in C, similar to what should be expected for a lower stability of the τ-phase.

**Figure 7:**
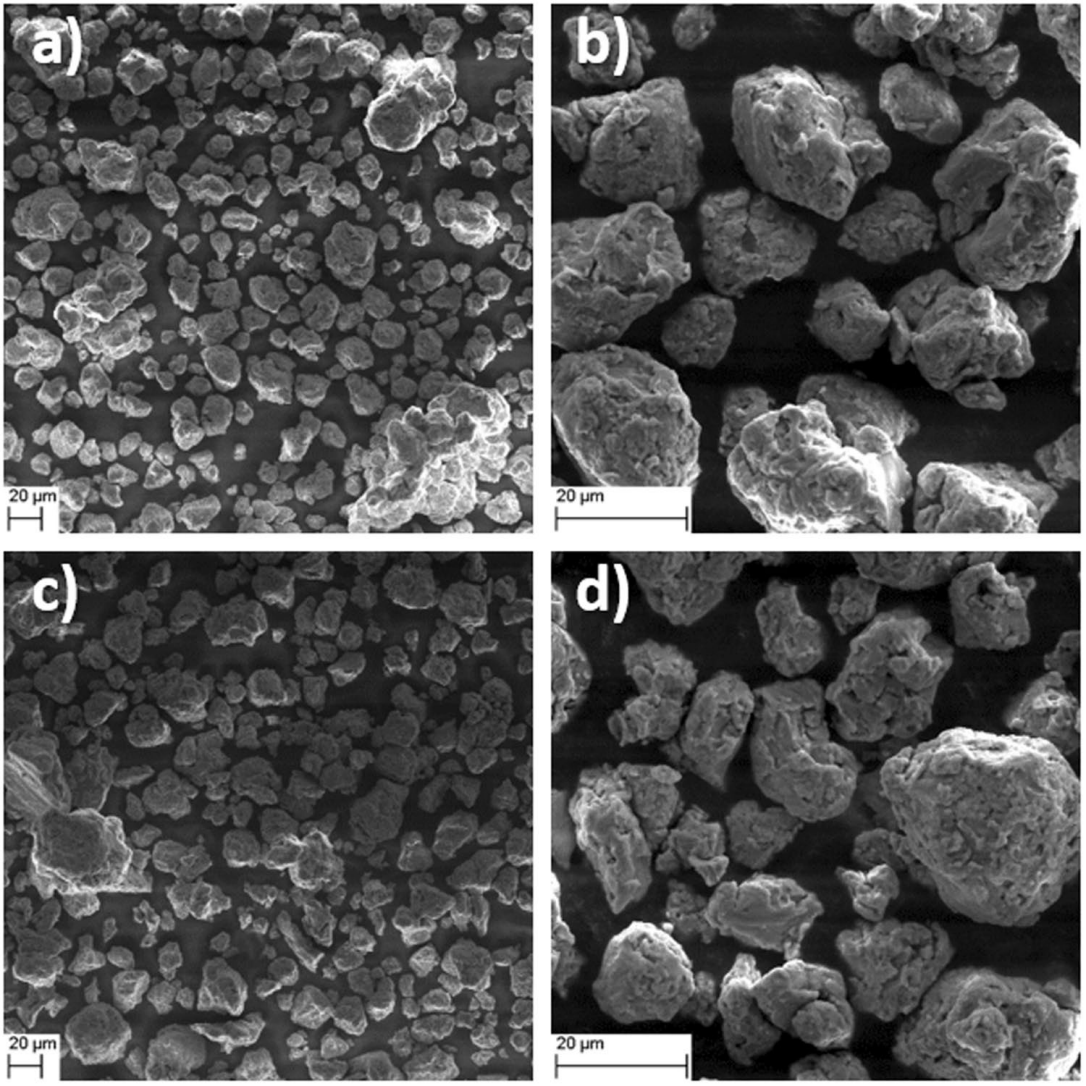
SEM images of (**a**,**b**) 2CM and (**c**,**d**) 4CM.

Cryo milling increases the potential capability of aligning the crystals along the easy magnetization axis, without the creation of sheet-like particles with random orientation, as previously observed when milling is done at ambient temperatures^25,28^. However, for this to be a viable route, single crystal grains (or particles with correspondingly aligned grains) are necessary. The particles yielded from cryo milling (see Fig. 7) are polycrystalline, which is disadvantageous to the hard magnetic properties of the samples, yielding coercivities lower than reported in some other studies, see e.g^29^.

**Figure 8:**
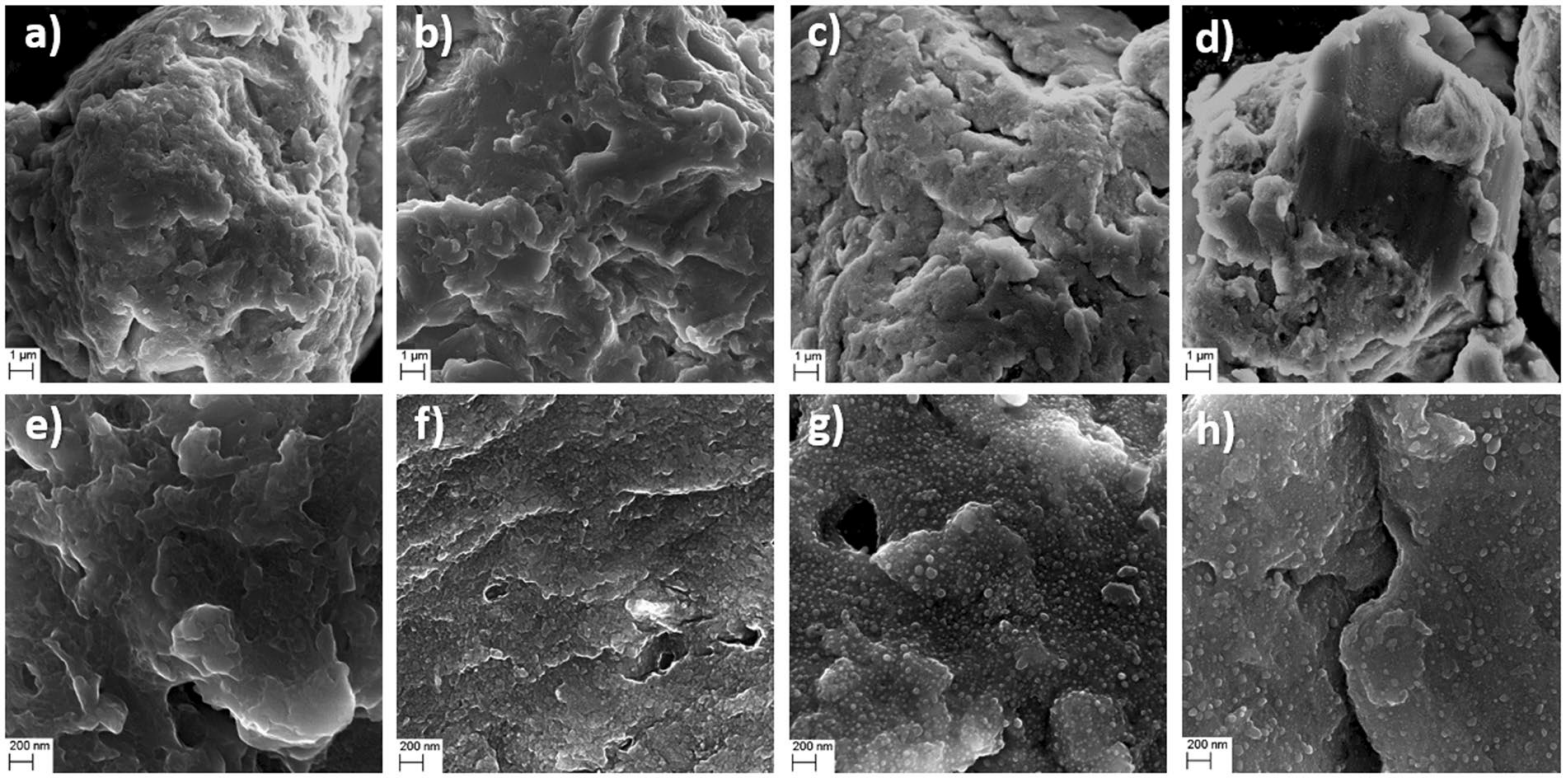
Observed surface morphologies for (**a**) 2CM, (**b**) 4CM, (**c**) 5 min flash heated 2CM, and (**d**) 1 min flash heated 4CM with observations (**e**–**h**) at higher magnifications for each sample, respectively.

From the SEM images in Fig. 8, it can be seen that the average particle size remains ~20 µm after both 2 hours and 4 hours of cryo milling. There could be a slight increase in surface and shape irregularity of the particles with time during cryo milling from 2 hours to 4 hours but it is not possible to determine statistically, although the surface smoothness seem to increase with longer milling time.

**Figure 9:**
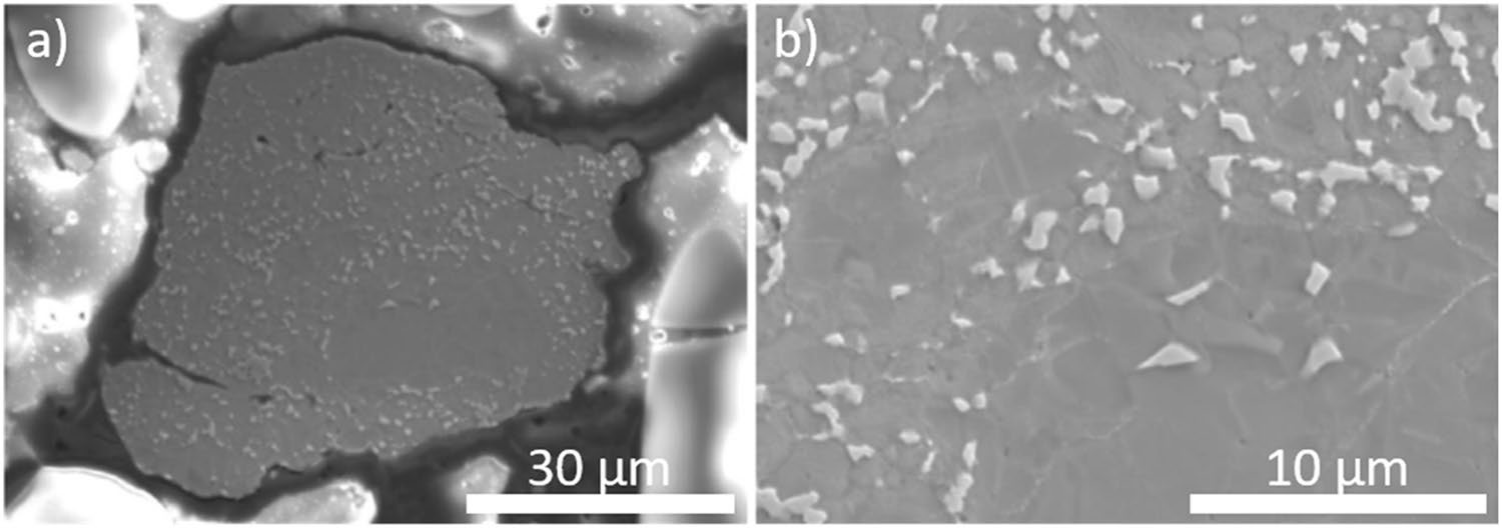
Observed microstructure in SEM at (**a**) low and (**b**) high magnifications after etching.

A closer view of the surface morphologies in Fig. 8 reveal a slight difference between 2CM (a and e) and 4CM (b and f) related to the surface roughness. The 2CM sample appears rougher on the µm scale compared to the 4CM sample (by visual inspection). However, at higher magnifications, the 4CM seems to have attained a rougher surface on the nm scale. Moreover, powder from the 2CM and the 4CM were flash heated and analyzed to reveal potential changes to the surface of the particles due to the heat treatment. Figure 8c reveal a surface with more rounded features at the µm compared to the 2CM sample (see Fig. 8a). The SEM images further reveal that the particles do not accumulate from cryo milling. At higher magnifications (see Fig. 8g) the surface exhibit new spherical features <100 nm in diameter. These features are, however, less pronounced, and fewer for the 4CM sample (see Fig. 8e) where only 1 min of flash heating at 900 °C has been used. From Fig. 8 it is therefore possible to discern a likely effect on the surface properties caused by the different flash heating treatments after the cryo milling process (*e*.*g*., the precipitation of spherical features <100 nm in diameter). In this case, the composition of the spherical features was not possible to determine easily due to the limit of the spatial resolution in the SEM for EDS analysis (~1–2 µm at 20 kV). In Fig. 9a and b, a polished and etched flash heated 4CM sample is shown at low, and high magnification, respectively. The white precipitates are found to be enriched in Al while the darker matrix region is higher in Mn.

### Magnetic properties characterization

**Figure 10:**
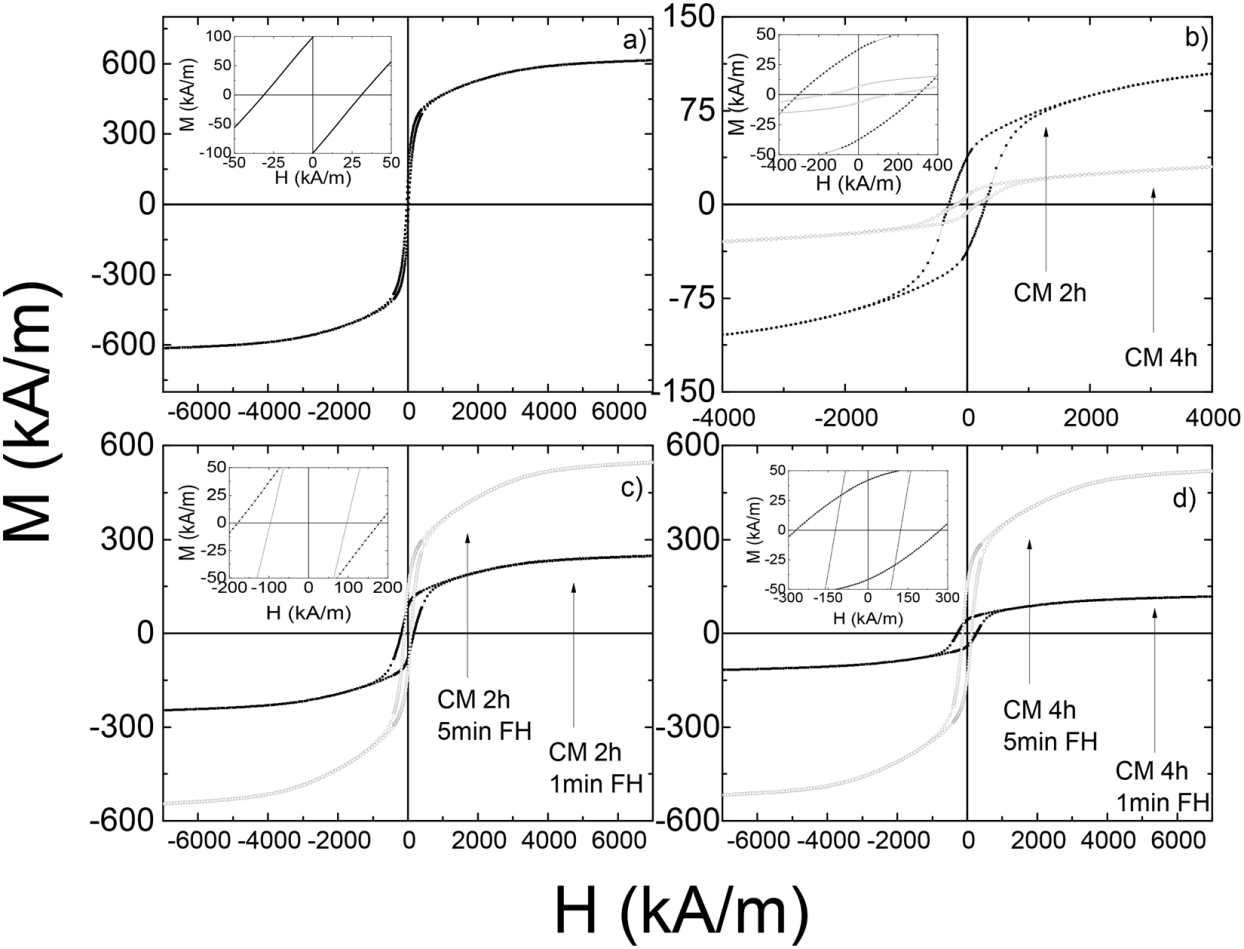
Magnetization versus magnetic field for (**a**) DS, (**b**) 2CM and 4CM, (**c**) 2CM flash heated 1 and 5 min and (**d**) 4CM flash heated 1 and 5 min.

The effect of the cryo milling process on the magnetic properties (*i*.*e*., *H*_*c*_ and *M*_*s*_) was evaluated from magnetization versus magnetic field measurements. From the magnetic hysteresis loop of the DS sample (cf. Fig. 10a) the coercive field is obtained as ${\mu }_{0}{H}_{c}\approx 40$ mT, while the value of the room temperature saturation magnetization ${M}_{s}\approx 614.8$ kA/m is close to its theoretical maximum value. Furthermore, the effect on the magnetic properties from cryo milling followed by flash heating (1 min, 5 min and 15 min) is illustrated by the *M* − *H* results for the 2CM and 4CM samples in Fig. 10b–d. Overall the magnetization is seen to decrease significantly with an increase in milling time. After 1 min of flash heating at 900 °C, the *M*_*s*_ for 2CM and 4CM only recovers to ~40% and ~19% of the original DS sample. The *H*_*c*_ for the 2CM and 4CM sample is, however, ~475% and ~750% higher than that of the original DS sample, respectively (see Table 2). Further increase of the flash heating time from 1 min to 5 min at 900 °C results in an *M*_*s*_ value ~88% and ~84% of the DS sample for the 2CM and 4CM samples, respectively. The *H*_*c*_ values for the 2CM and 4CM samples are however, only ~200% and ~300% higher than that of the original DS sample after 5 min of flash heating at 900 °C. Moreover, heating for 15 min only resulted in minor changes as seen in Table 2.

**Table 2:** Summary of the magnetic properties for the DS sample and the flash heated 2CM and 4CM samples.

	***M***_*s*_ (kA/m)	***M***_*s*_ (µ_B_/f.u.)	***M***_***r***_ (kA/m)	Mr/Ms (N = 1/3)	*μ*_0_***H***_*c*_ (kA/m)
Drop synthesized					
(Mn_0.55_Al_0.45_)_100_C_2_	614.8	1.84	120.4	42.1	32
Cryo milled 2 h (2CM)	104.4	0.31	37.4	37.4	302
Flash heated 1 min	248.4	0.74	81.1	37.1	183
Flash heated 5 min	542.5	1.62	133.6	34.9	95
Flash heated 15 min	527.9	1.58	132.1	35.0	103
Cryo milled 4 h (4CM)	29.8	0.09	6.8	24.0	176
Flash heated 1 min	117.6	0.35	41.9	36.4	270
Flash heated 5 min	515.8	1.54	148.4	38.3	127
Flash heated 15 min	515.2	1.54	142.8	36.7	119

The *M*_*s*_-values expressed as µ_B_/f.u. have been calculated for Mn_1.1_Al_0.9_C_0.02_ using the cell volume 27.96 Å^3^.

The M_r_/M_s_ ratio of the samples are less than the expected 50% when taking into account the demagnetizing field. The demagnetizing factor was approximated to N = 1/3 (sphere) according to the approach by Bleany and Hull for spherical particles. The low values M_r_/M_s_ of <50% can be attributed to the formation of epsilon phase and the unindexed phase followed through CM and through the synthesis (see Fig. 5a). This also explains the lower magnetization values (see Fig. 10 and Table 2) compared to the DS samples. It is also possible that the loosely packed powder should have a deviation from the assumed demagnetizing factor.

The highest M_s_ value obtained here, 614.8 kA/m, is near what has been reported previously for other C- doped samples; 639.1 kA/m^30^, 596.7 kA/m^14^, 587.6 kA/m^31^, 581.4 kA/m^25^, 557 kA/m^32^ and 475 kA/m^33^. Higher M_s_ values of 650 kA/m have been reported for samples without C-doping^30^. Wei *et al*. also reported coercivities as high as 500 mT, but then the saturation magnetization was reduced to roughly 100 kA/m, which is lower than that we obtain when flash heating the samples for a mere 1 min. Since the experimental method used by^29^ was melt spinning, creating ribbons of the t-phase, followed by ball milling, they were in the favorable position to obtain nanometer-sized grains and particles containing far less grains as compared with our particles. Our particles are some tens of micrometer in size containing thousands of randomly oriented grains. Alignment of the grain easy axes by applying a magnetic field is thus not possible in our case, while Wei *et al*. indicate that alignment in a field of 1 T improved the hard magnetic properties. The M_s_ obtained here is also close to the theoretical maximum of 690 kA/m^8^.

## Discussion

Analysis of the structural, microstructural and magnetic data obtained from both drop synthesized and cryo milled Mn_0.55_Al_0.45_C_0.02_ samples provides insight into the effect of processing on the magnetic properties. From the XRPD and the NPD (see Fig. 1a) data of the DS sample synthesized by cooling from the melt at 1400 °C, a single phase τ-phase can be clearly identified. However, from the EDS mapping (see Fig. 6) and the LOM (see Fig. 4) images, a crystalline region can be identified with higher Mn and C content in contrast to the matrix region at the boundaries, which is enriched in Al (see Fig. 6c). These Al rich regions are also observed as white regions after polishing and etching 900 °C flash heated 4CM (see Fig. 9). These EBSD mapping of the regions (see Fig. 5a) clearly identify the crystalline phase as the τ-phase (blue) and the Al-enriched nano-crystalline X-ray amorphous Al-enriched matrix region as mainly the ɛ-phase (green) with some minor impurities of β-Mn (yellow). The solid solubility of Al in β-Mn is very high and could explain why these inclusions were not observed in the EDS mapping. The microstructural analysis clearly shows that the full transformation of ɛ → τ has not been achieved, despite the indication of a single phase material from the XRPD and NPD patterns.

Based on the microstructure, three explanations for the observations are suggested: 1) the total volume of the ɛ-phase is below the detection limit of the XRPD; or 2) the regions are too small to be detected by XRPD and NPD; and 3) large strains on the regions broadens the impurity peaks to a degree, not easily detectable from the background in XRPD and NPD. The second explanation can partly be excluded based on the observations of 1–50 µm large domains in both LOM and ESBD (see Figs 4 and 6, respectively), EBSD shows that the total volume of the ɛ-phase should be enough to be detected (~15%, the amount of matrix phase was however less in other regions of the sample). It is thus suggested that strain in combination with the very small grains in the matrix region is the most probable explanation for the XRPD and ND results. Although, the microstructural observations are from 2D and not 3D, one can observe the inclusion of much smaller grains within the matrix phase both in the LOM and SEM observations (see Figs 4 and 6, respectively). The small grains <1 µm are believed to be formed during the ɛ → τ transformation, where twinning formation is highly prevalent^7^. These grains will in addition be highly strained and most likely contribute to the peak broadening observed from XRPD and NPD (see Fig. 1a,b).

From the magnetic properties (see Table 2) of the DS sample, the *M*_*s*_ value ~614.8 kA/m, it can be concluded that the ɛ → τ transformation is close to complete. The obtained *M*_*s*_ value allows us to calculate a theoretical value for ${(BH)}_{max}={\mu }_{0}{M}_{s}^{2}/4\approx 120$ kJ/m^3^. The theoretical (*BH*)_*max*_ can only be reached if all single magnetic domain particles have their easy magnetization directions aligned along one common direction. From the observations in LOM and SEM (see Figs 4 and 6, respectively), the grains are randomly oriented and too large to be single magnetic domains, and will therefore lead to a hysteresis loop more similar to a “soft” magnet with a low *H*_*c*_ (see Fig. 10).

Cryo milling the DS sample resulted in rounded particles with ~20 µm in diameter after both 2 and 4 hours of cryo milling. Longer milling time than 2 hours did not appear to decrease the particle size much further, but rather to some extent make the particles less regular on the µm scale (see Fig. 8a,b), despite the smoother surface observed. From the XRPD and the NPD results the crystal structure appears to become more strained as the peaks appear to broaden with increasing milling time (see Fig. 1c–f), which gradually introduces more and more defects in the material.

In particular, all the main peaks in the NPD pattern after 4 hours of cryo milling disappear into the background (see Fig. 1f), while the same peaks are broadened in the XRPD data. Furthermore, the peaks in the XRPD data (see Fig. 1e) clearly show the existence of a long range atomic ordering. It is thus important to note, however, that the weak reflections related to the *L1*_0_ structure (at 2.28 and 3.48 Å^−1^) become weaker after 2 hours of milling (see Fig. 1c), and disappear completely after 4 hours of milling (see Fig. 1e). From the SEM images (see Fig. 7), the particle size was kept at ~20 µm even for the 4CM sample and should not affect the peak width significantly. These results indicate that the initial strong ordering between Mn and Al in the τ-phase may have been disturbed by *e*.*g*., defect generation from the ball milling procedure, rather than being a consequence of a decrease in particle size. The increased inter-site mixing between Mn and Al on the separate lattice points in the *L1*_*0*_ structure is confirmed by Rietveld refinement of the Mn and Al occupancies on the 1a and 1d sites (see Table 1). In addition, the nuclear scattering amplitudes of Mn and Al are nearly equal, but with different signs (*i*.*e*., coh b_Mn_ = −3.73 fm and coh b_Al_ = 3.449 fm^34^). It is therefore likely that a large inter site mixing of Mn and Al will lead to nearly zero observable scattering amplitudes in the NPD data. It can therefore be concluded that, prolonged cryo milling is detrimental for the ordering of the Mn and Al atoms, which in turn will degrade the magnetic properties (*i*.*e*., *M*_*s*_). This is further confirmed by M-H measurements of the DS, 2CM and the 4CM samples (see Fig. 10 and Table 2).

To regenerate the order between Mn and Al in the *L1*_*0*_ - structure by decreasing strain and other defects in the material, flash heating was used. From Fig. 2 it is clear that the 2CM and the 4CM samples regains a large part of the Mn and Al ordering as the diffraction pattern is nearly fully restored. However, the occupancy on the Mn and Al sites was not possible to refine from XRD data alone and were thus fixed to the values of the DS sample (see Table 1). From SEM observations (see Fig. 7) no clear change in the particle size is observed, however, to some extent the particles have lost the distinct smooth surfaces on the µm scale (see Fig. 7c), which can be ascribed to the heat exposure during the flash heating. At higher magnifications, the spherical precipitates <100 nm observed on the surface and inside the particles (see Fig. 7g,h) appear to form with increased flash heating time. These precipitates are most likely a low melting point phase or Mn that starts to evaporate. Experimentally we have observed a slight greyish discoloring of the inside of the quartz ampule, which may be ascribed to the Mn evaporation.

The greyish discoloring appear to significantly increase for smaller particle size, and therefore we believe the flash heating time should be shortened for the cryo milled samples compared to *e*.*g*., manually grinded samples with larger grain sizes. In addition, this behavior also indicates that the time required at high temperature (*e*.*g*., 900 °C) to regain the initial *L1*_*0*_ phase with little mixing between the Mn and Al atoms, decreases with decreasing particle size. This may have important implications for the processing of MnAl based materials, for which the powder needs to maintain a narrow particle size distribution with sub-micron mean particle size to optimize e the permanent magnet properties.

Our *in situ* XRPD studies supports the possibility to cycle between the ɛ-, and the τ-phase (see Fig. 3) at a cooling rate of 50 °C/min. Earlier reports on the MnAl(C) system shows that a cooling rate ΔT < 87 °C/min is crucial for the ɛ → τ transformation^35^, and recently Shao *el al*. also reported the one step preparation of pure τ-phase MnAl^36^. Furthermore, we demonstrate the flexibility of this transformation by its utilization in the flash heating procedure to recrystallize the τ-phase within the small grains and thus bypassing the formation of Mn and Al rich equilibrium phases^13,36^. Besides, the XRPD patterns of the 5 min and 15 min flash heated samples (see Fig. 2), clearly confirms the recrystallization of the τ-phase after exposure to heat at 900 °C. However, after the flash heating, detectable traces <10 at.% of Al-rich (Cr_5_Al_8_/Al_8_Mn_5_ type^37^) phases are observed.

It is well known that magnetic domain wall pinning can be enhanced by defects and impurities grain boundaries or within grains in case of multi-domain grains^7,33^. The coercivity for the longest milling time (4 hours) is also the highest, while its magnetization, *M*_*s*_ is the lowest. Similar inverse relationship has been observed previously for other types of materials^38^. However, it appears that the flash heating restores a much larger percentage of the *M*_*s*_ than is lost from the *H*_*c*_ for the 2CM and 4CM samples. This indicates that the Mn and Al site disorder within grains should be minimized to maximize the *M*_*s*_. The higher *H*_*c*_ values are likely due to a maintained roughness on the nm level at the particle surfaces in addition to the large amount of defects and potential secondary phases (*e*.*g*., the spherical precipitates). The cryo milling procedure followed by flash heating can therefore be thought of as a viable way to simultaneously enhance the *H*_*c*_ and *M*_*s*_ values. In particular, the XRPD data clearly indicates the recrystallization of the τ-phase inside the particles after the flash heating (as evidenced by strong sharp peaks), indicating that the ordering between Mn and Al has been restored. This effect is also reflected in the increased *M*_*s*_ values for the cryo milled and flash heated powders (see Table 2). Moreover, the small particle size after cryo milling likely contributes to the recrystallization of fewer but larger grains of the τ-phase (as indicated by the reduction in FWHM compared with the DS sample) with improved crystallographic alignment; which in combination with surface impurities and nm sized precipitates (see Fig. 8) likely lead to a maintained large coercivity.

## Conclusions

In conclusion, a new way to process MnAl-based alloys has been developed. By using a combination of cryo milling and flash heating, a high purity τ-phase MnAl material can be obtained. The flash heating process allows control of the microstructure and minimizes grain growth, while keeping a high phase purity of the material. Compared with conventional heating, the flash heating can bypass the formation of unwanted equilibrium phases, thus keeping a high saturation magnetization of the post milled material. The obtained results shows the possibility of controlling microstructure and magnetic properties independently, enabling a way forward to producing high performance MnAl-based permanent magnets.

## Methods

### Sample synthesis

The Mn_0.55_Al_0.45_C_0.02_ alloy ingot was synthesized by the drop synthesis process^39^ at 1400 °C under argon atmosphere, the total mass of the sample was 20 g. High purity raw materials were used, Mn (Institute of Physics, Polish Academy of Sciences, purity 99.999%) C (Highways international, 99.999%) and Al (Gränges SM, purity 99.999%). First the Al metal was heated up and melted with carbon in the crucible at 1000 °C. Small pieces of Mn metals were subsequently dropped into the melted Al-C alloy, then the eddy current power was increased to enable the Mn pieces to react with Al-C liquid alloy immediately. The melt was kept at 1400 °C for 10 minutes to ensure that the Mn-Al-C liquid forms a homogeneous solution. The Mn-Al-C alloy was cooled down to room temperature by natural cooling.

### Cryo milling process

Cryogenic milling was performed at liquid nitrogen temperatures using a SPEX Freezer/Mill 6770. The starting material was placed in a specially designed stainless steel vial with a stainless steel cylindrical impactor. The mass ratio between the impactor and the powder was 30:1. Before the milling was started, the vial was allowed to cool down for 30 min in the liquid nitrogen bath of the Freezer/Mill. The milling was then carried out at an impact frequency of 30 Hz for a total of 2 or 4 hours. Each milling run consisted of 5 minutes milling and 3 minutes pause cycles.

### Diffraction

X-ray powder diffraction (XRD) was performed at a Bruker Twin-Twin diffractometer, with a Cu double Kα radiation (λ_1_ = 1.540600 Å, λ_2_ = 1.544390 Å). The neutron powder diffraction was carried out at the JEEP-II reactor in Kjeller, Norway using the PUS instrument with monochromatized neutrons (λ = 1.556 Å). The crystal structure and phase analysis were treated by FullProf software through the Rietveld method^40^. The peak shape of the diffraction pattern was characterized by the Thompson-Cox-Hastings pseudo-Voigt function.

The phase transition behaviors of 2 and 4 hours cryo milled Mn_0.55_Al_0.45_C_0.02_ samples when heated and cooled at different rate was investigated by *in situ* synchrotron X-ray diffraction at the P02.1 beamline at PETRA III (λ = 0.207 Å). The powder cryo milled Mn_0.55_Al_0.45_C_0.02_ samples were loaded in a single crystal sapphire tube, the tube was wounded by Kanthal wire and heated up to 900 °C in vacuum (50 °C/min), dwelled at 900 °C for 5 minutes then cooled (50 °C/min) to room temperature. The temperature was monitored by a K type thermocouple insert from one side of the sapphire tube with close contact to the sample (inside the sapphire tube). The sample to detector distance and X-ray beam wavelength was determined and calibrated by the NIST LaB_6_ standard sample. The X-ray diffraction patterns were recorded by a PerkinElmer XRD1621 fast area detector. The diffraction patterns of 2D pictures were transformed to 1D diffractograms by the Fit2D program.

### Flash heating

For the flash heating process, samples were first put into Al_2_O_3_ crucibles, the crucibles were sealed in evacuated quartz tubes. Then, the ampoules were transferred to a pre-heated resistance furnace and “flash heated” at 900 °C for 1 minutes, 5 minutes, 15 minutes respectively, followed by air cooling.

### Magnetic property characterization

Powder samples were placed in gelatin capsules with varnish. The capsule and varnish together contribute a paramagnetic moment at 300 K and account for <0.01% of the saturation magnetic moment at 9 T. Samples were measured at room temperaure in a Physical Properties Measurement system from Quantum Design equipped with a 9 T superconducting magnet or a MPMS from Quantum Design. Magnetization in SI units and µ_B_ were calculated from the sample weight and using the lattice parameters obtained from the XRD/NPD refinements. M_s_ was obtained from the magnetization at maximum magnetic field (7200 kA/m for most samples and 4000 kA/m for one of the samples). Experimental densities obtained from XRD and NPD were used when calculating the volumetric magnetization.

### Microstructure

Pieces from the drop synthesized sample with the nominal composition Mn_0.55_Al_0.45_C_0.02_ were mounted in a conducting polymer resin and grinded and polished down to a surface roughness of 1 µm. The flash heated 4CM sample was analyzed with a scanning electron microscope equipped with the Field Emission Scanning Electron Microscopy (FE-SEM) (Hitachi SU6600) and X-ray energy dispersive spectroscopy (EDS) (Bruker EDX XFLASH 5010). The same procedure was used to prepare the sample for LOM and EBSD analysis, but, without the etching. The LOM analyses were performed using a Zeiss Axio Imager M2m microscope in bright field (BF) mode and in circular differential interference contrast (C-DIC) mode. The EBSD analysis was performed using a Zeiss Merlin HR scanning electron microscope with an Oxford Nordlys Max EBSD detector. The microscope was operated at 25 kV accelerating voltage and 20 nA probe current. Phase mapping was made using the AztecHKL software from Oxford instruments. The phase identifications from the obtained Kikuchi patterns were made using crystallographic information from the cell parameters of the ɛ-, β-, γ_2_-, and the τ-phases obtained from Pearson’s Crystal Data. The final phase color image obtained from EBSD was smoothed to remove noise and zero solution points in the image. Cryo milled powder samples were fixed on separate aluminum stubs with conducting carbon glue, and investigated by SEM and EDS using a Zeiss Gemini 1550 scanning electron microscope.

### Data availability

All the data that provided in this manuscript are available to the general readers.

## Electronic supplementary material

Supplementary information
