# Supplementary material for: Structural, microstructural and magnetic evolution in cryo milled carbon doped MnAl
Source: Sci Rep. 2018 Feb 6;8:2525. doi: 10.1038/s41598-018-20606-8 (PMC5802788; doi:10.1038/s41598-018-20606-8)
Supplement: Supplementary file 1 — Supplementary information [file 41598_2018_20606_MOESM1_ESM.pdf]

Structural, microstructural and magnetic evolution in cryo milled carbon doped MnAl

Hailiang Fang[1], Johan Cedervall[1], Daniel Hedlund[2], Samrand Shafeie[1], Stefano Deledda[4], Fredrik Olsson[5], Linus von Fieandt[1], Jozef Bednarcik[3], Peter Svedlindh[2], Klas Gunnarsson[2] and Martin Sahlberg[1]

1.Department of Chemistry – Ångström Laboratory, Uppsala University, Box 538, 75121 UPPSALA, Sweden

2. Department of Engineering Sciences, Uppsala University, Box 534, 751 21 Uppsala, Sweden

3. Deutsches Elektronen Synchrotron DESY, Notkestrasse 85, D-22603 Hamburg, Germany

4. Institute for Energy Technology Instituttveien 18NO-2007 Kjeller, Norway.

5, Höganäs AB, Bruksgatan 35, 263 33 Höganäs

Supplementary information

Table SI1. Reliability factors for the combined refinements. R-values are for the τ-phase.

	$R_{X\text{-rays}}$	$R_{neutron}$	R_{mag}	χ^2
As-prep	1.43	3.49	4.00	0.8917
CM 2h	6.98	9.47	6.50	2.980
CM 4h	5.15	-	-	1.837

Table SI2. Reliability factors for the refinements of flash heated samples. R-values are for the τ-phase.

	$R_{X\text{-rays}}$	χ^2
CM2h 5min	15.2	5.315
CM2h 15min	14.9	4.555
CM4h 5min	12.3	6.235
CM4h 15min	22.2	5.179

Figure SI1

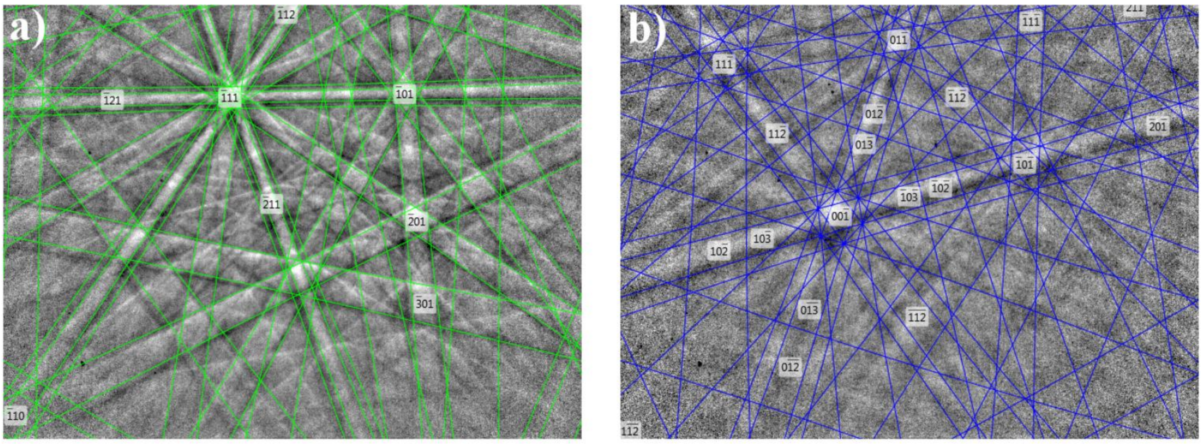

Selected EBSD diffraction patterns of the τ- and ε-phase are shown in Figure a and b, respectively.
